# Upregulated long non-coding RNA AFAP1-AS1 expression is associated with progression and poor prognosis of nasopharyngeal carcinoma

**DOI:** 10.18632/oncotarget.4057

**Published:** 2015-05-09

**Authors:** Hao Bo, Zhaojian Gong, Wenling Zhang, Xiayu Li, Yong Zeng, Qianjin Liao, Pan Chen, Lei Shi, Yu Lian, Yizhou Jing, Ke Tang, Zheng Li, Yanhong Zhou, Ming Zhou, Bo Xiang, Xiaoling Li, Jianbo Yang, Wei Xiong, Guiyuan Li, Zhaoyang Zeng

**Affiliations:** ^1^ Hunan Cancer Hospital and The Affiliated Cancer Hospital of Xiangya School of Medicine, Central South University, Changsha, Hunan, China; ^2^ The Key Laboratory of Carcinogenesis of the Chinese Ministry of Health and The Key Laboratory of Carcinogenesis and Cancer Invasion of the Chinese Ministry of Education, Cancer Research Institute, Central South University, Changsha, Hunan, China; ^3^ Hunan Key Laboratory of Nonresolving Inflammation and Cancer, Disease Genome Research Center, The Third Xiangya Hospital, Central South University, Changsha, Hunan, China; ^4^ Department of Oral and Maxillofacial Surgery, The Second Xiangya Hospital, Central South University, Changsha, Hunan, China; ^5^ Department of Laboratory Medicine and Pathology and Masonic Cancer Center, University of Minnesota, Minneapolis, Minnesota, United States of America

**Keywords:** long non-coding RNA (LncRNA), AFAP1 antisense RNA1 (AFAP1-AS1), nasopharyngeal carcinoma (NPC), metastasis, prognosis

## Abstract

Altered expression of long noncoding RNAs (lncRNAs) associated with human carcinogenesis. We performed a cDNA microarray analysis of lncRNA expression in 12 cases of nasopharyngeal carcinoma (NPC) and 4 non-tumor nasopharyngeal epitheliums. One lncRNA, actin filament associated protein 1 antisense RNA1 (AFAP1-AS1), was identified and selected for further study. AFAP1-AS1 expression was upregulated in NPC and associated with NPC metastasis and poor prognosis. *In vitro* experiments demonstrated that AFAP1-AS1 knockdown significantly inhibited the NPC cell migration and invasive capability. AFAP1-AS1 knockdown also increased AFAP1 protein expression. Proteomic and bioinformatics analyses suggested that AFAP1-AS1 affected the expression of several small GTPase family members and molecules in the actin cytokeratin signaling pathway. AFAP1-AS1 promoted cancer cell metastasis *via* regulation of actin filament integrity. AFAP1-AS1 might be a potential novel marker that can predict cancer patient prognosis and as a potential therapeutic target for NPC.

## INTRODUCTION

In Southeastern Asia, nasopharyngeal carcinoma (NPC) is a unique disease with significantly different risk factors, pathogenesis, clinical behaviors and treatment options than other head and neck cancers [1]. Epstein-Barr virus, environmental influences and heredity each play important roles in NPC development [2-6]. Like most other human cancers, NPC development is a multi-step process that involves oncogene activation and tumor suppressor silencing [7-11]. Many genetic and epigenetic alterations in NPC have been reported [12]; however, the precise molecular mechanisms underlying NPC development and progression remain unclear. Therefore, further investigation and molecular profiling of NPC is necessary to improve understanding of NPC pathogenesis and to develop novel treatment strategies and biomarkers for early detection or the prediction of NPC progression, treatment response or prognosis.

Long noncoding RNA (lncRNA) regulates gene transcription and post-transcriptional regulation and dysregulated LncRNA expression plays a crucial role in human carcinogenesis [13]. Thus, in the current study, we focused on the role of lncRNAs in NPC. LncRNAs are non-protein-coding transcripts that are ≥ 200 nucleotides in length, and accumulating evidence indicates that they participate in many physiological processes by modulating gene expression at the epigenetic, transcriptional and posttranscriptional levels. Dysregulated lncRNA expression has been reported in a variety of human cancers, including nasopharyngeal carcinoma (NPC), lung, breast and colorectal cancers. LncRNAs appear to participate in all stages of cancer development, including tumor initiation, progression and metastasis. To date, more than 50,000 lncRNAs have been reported in the human genome; however, the function of most of these lncRNAs remains unknown [13-18].

We preformed gene expression profiling analysis using gene microarray technology because this method is a proven and powerful tool to find altered gene expression in paired samples, such as normal and cancerous tissues. In this study, we profiled the genes expressed in 12 NPC and 4 non-tumor nasopharyngeal epithelial (NPE) biopsies using Affymetrix HG_U133 Plus 2 arrays. We then confirmed our data for lncRNA dysregulation using one previously published online NPC datasets (GSE12452) [19]. One lncRNA, the actin filament associated protein 1 antisense RNA 1 (AFAP1-AS1, NCBI accession number: NR_026892, Affymetrix probe set: 223779_at) was significantly overexpressed in NPC. Then, we examined NPC expression in 112 paraffin-embedded NPC tissue specimens and performed *in vitro* knockdown experiments targeting AFAP1-AS1 expression in NPC cells to assess the changes in tumor cell behavior when AFAP1-AS1 expression is lost.

This study represents a significant step forward in understanding the importance of lncRNAs in NPC, and provided a novel insight concerning the role of AFAP1-AS1 in the development and progression of NPC. Future studies based on these findings may lead to discover novel NPC biomarkers or targeted therapies.

## RESULTS

### AFAP1-AS1 upregulation is associated with poor prognosis in NPC

**Figure 1:**
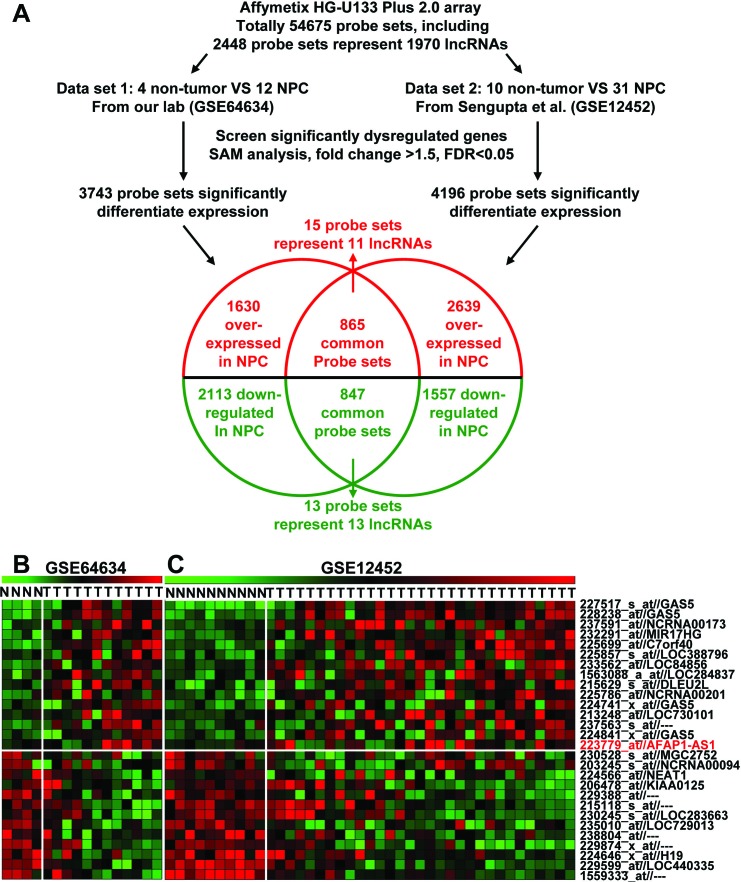
Dysregulated lncRNA expression analysis using two independent NPC cohorts and cDNA microarray analysis **A.** Schematic overview of the workflow used to identify and validate dysregulated lncRNAs in two NPC microarray data cohorts. **B.** Heatmap of 28 dysregulated probe sets representing 24 lncRNAs mined from our own NPC dataset (GSE64634). **C.** Heatmap of 28 dysregulated probe sets representing 24 lncRNAs mined from the GSE12452 data set.

We first profiled differentially expressed genes in NPC and normal tissues (GSE64634) and compared our results with a similar previous study (GSE12452) [19]. These two NPC gene expression cohorts were used with the Affymetrix HG U133 Plus 2.0 gene chip platform. We found 4196 differentially expressed genes in the GSE12452 dataset and 3743 in the GSE64634 dataset. Based on NetAffx, Refseq and Ensembl non-coding RNA annotations, we identified 28 overlapping probe sets, representing 24 lncRNAs that were differentially expressed in NPC when compared to normal nasopharyngeal epithelia. Of these 24 lncRNAs, 11 were upregulated and 13 were downregulated (Figure 1 and Supplemental Table S1).

**Figure 2:**
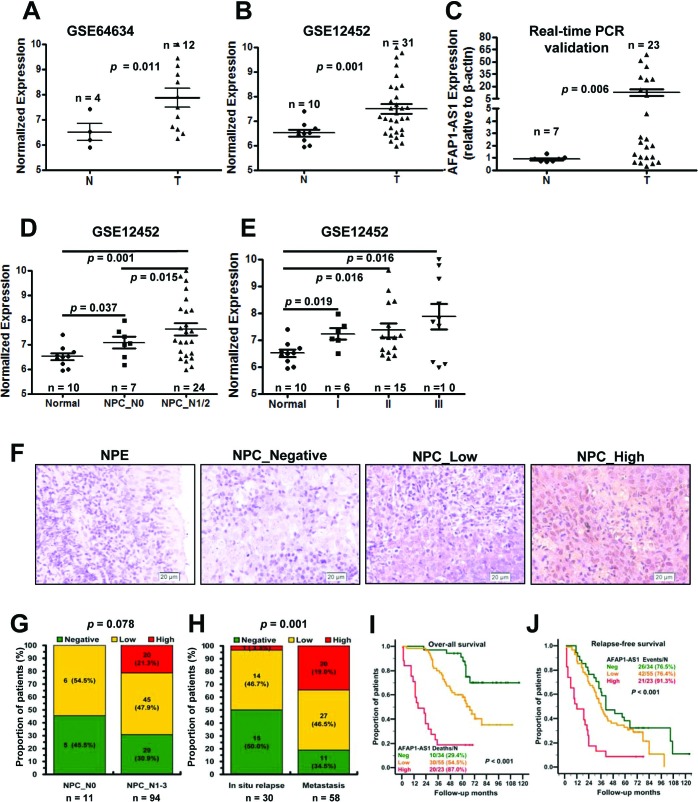
Upregulated AFAP1-AS1 was associated with tumor metastasis and poor prognosis **A.** AFAP1-AS expression, as measured by Affymetrix Microarray, was upregulated in NPC biopsies (T, *n* = 12) when compared with non-tumor NPE tissues (N, *n* = 4). **B.** Upregulated AFAP1-AS expression was confirmed in NPC biopsies (T, *n* = 31) compared with non-tumor NPE tissues (N, *n* = 10). **C.** AFAP1-AS expression in non-tumor NPE tissues (N, *n* = 7) and NPC biopsies (T, *n* = 23) was validated in another cohort of NPC samples using qRT-PCR. **D.** AFAP1-AS1 expression was associated with lymph node metastasis in the GSE12452 dataset (normal: non-tumor NPE; NPC_N0: NPC biopsies without lymph node metastasis; N1/2: NPC biopsies with lymph node metastasis). **E.** AFAP1-AS1 expression was associated with clinical disease stages in the GSE12452 dataset (normal: non-tumor NPE; I, II or III: NPC biopsies with clinical stage I, II or III disease). **F.** AFAP1-AS1 expression measured by *in situ* hybridization in paraffin embedded non-tumor NPE (N) and NPC biopsies (T). Representative cases of non-tumor NPE (N) and NPC biopsies with negative (*n* = 34), low (*n* = 55) or high (*n* = 23) AFAP1-AS1 staining are shown. **G.** Proportion of NPC patients with AFAP1-AS1 expression with lymph node metastasis (N0, *n* = 11; N1/2, *n* = 97, *p* = 0.078). **H.** Proportion of NPC patients with AFAP1-AS1 expression and *in situ* relapse (*n* = 30) or distant metastasis (*n* = 58, *p* = 0.001). **I.** and **J.** Overall survival and relapse-free survival analysis of patients with negative, low and high AFAP1-AS1 staining using a Kaplan-Meier curve, *p* < 0.001. T, tumor; N, non-tumor tissues.

Among the differentially expressed lncRNAs, AFAP1-AS1 was highly expressed in the NPC samples of both datasets (Figure 2A and 2B). Therefore, we next validated AFAP1-AS1 expression in another cohort of NPC samples using qRT-PCR. AFAP1-AS1 was highly expressed in 23 NPC samples when compared with 7 non-tumor nasopharyngeal epithelium samples (Figure 2C, *p* = 0.006). High AFAP1-AS1 expression was associated with a number of clinicopathological parameters in the GSE12452 dataset, including lymph node metastasis and TNM stage (*p* < 0.05; Figure 2D and 2E).

We next assessed AFAP1-AS1 expression in paraffin embedded NPC samples *via in situ* hybridization and found that AFAP1-AS1 expression was upregulated in NPC samples when compared with non-tumor NPE samples (69.64%, 78/112 vs. 20%, 2/10; *p* < 0.001; Figure 2F). We then analyzed AFAP1-AS1 expression for associations with clinicopathological parameters, such as gender, age, smoking, histological type, pathological stage, tumor size (T stage), lymph-vascular invasion (N stage) and relapse (Supplemental Table S2). The data indicated that AFAP1-AS1 expression was positive associated with distant tumor metastasis (*p* = 0.001, Figure 2H) and had a non-significant association with advanced tumor stage (*p* = 0.078, Figure 2G). High AFAP1-AS1 expression was also associated with poor overall survival (*p* < 0.001, Figure 2I) and poor relapse-free survival (*p* < 0.001, Figure 2J).

### AFAP1-AS1 knockdown suppressed tumor cell migration and invasion

**Figure 3:**
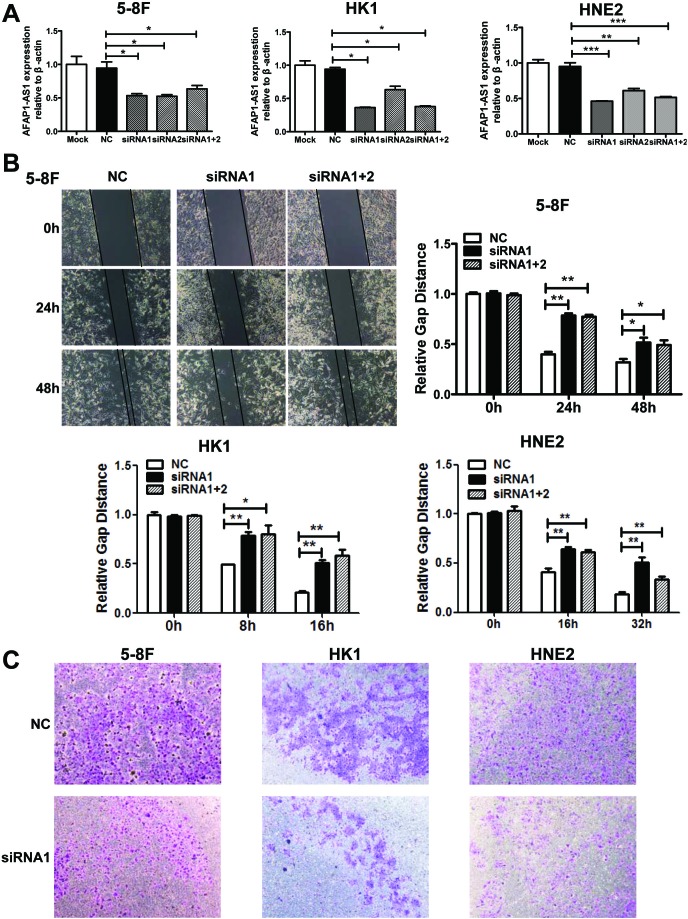
AFAP1-AS1 knockdown suppressed tumor cell migration and invasion *in vitro* **A.** siRNA knockdown of AFAP1-AS1 expression. siRNA1, siRNA2 and siRNA1+2 dramatically suppressed AFAP1-AS1 expression at the RNA level when compared with Mock and the scrambled control-siRNA (NC) in 5-8F, HK1 and HNE2 cells by qRT-PCR. **B.** AFAP1-AS1 knockdown inhibited 5-8F, HK1 and HNE2 cell migration. Cells were grown and transfected with AFAP1-AS1 siRNA or scramble siRNA for 24 h and then subjected to the wound healing assay at 0, 24 and 48 h (upper left panel). The data are summarized as the width ratio of migratory inhibition. Control siRNA treated cells efficiently migrated into the gap, while AFAP1-AS1 siRNA treated cells uniformly displayed significantly impaired wound closure (upper right panel and lower panel). **C.** AFAP1-AS1 knockdown inhibited tumor cell invasion as measured by Transwell Matrigel penetration assay. 5-8F, HK1 and HNE2 cells were grown and transfected with AFAP1-AS1 siRNA, or control siRNA for 36 h and then subjected to a Matrigel invasion assay. The graph summarizes the data from three independent experiments. *p < 0.05, **p < 0.01, ***p< 0.001.

To investigate the function of AFAP1-AS1 in cancer, we knocked AFAP1-AS1 expression down using two AFAP1-AS1 targeting short interfering RNAs (siRNAs) in three NPC cell lines 5-8F, HK1, and HNE2. The results demonstrated that both siRNA oligonucleotides could efficiently knock AFAP1-AS1 expression down by at least 60% in each of these cell lines (Figure 3A).

**Figure 4:**
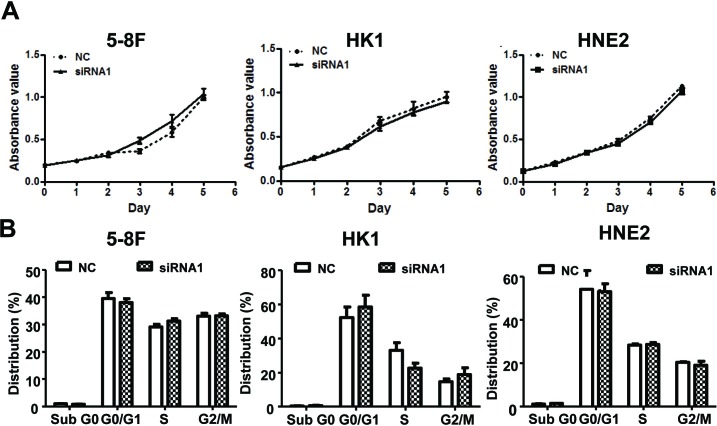
AFAP1-AS1 knockdown didn't affect cell viability cell cycle distribution and apoptosis *in vitro* **A.** Cell viability assays using 5-8F, HK1 and HNE2 cells. Cells were grown and transfected with AFAP1-AS1 siRNA or control siRNA and then subjected to cell viability MTT assays. AFAP1-AS1 knockdown had no effect on cancer cell viability. **B.** Cell cycle distribution and apoptosis detection. 5-8F, HK1 and HNE2 cells were grown and transfected with AFAP1-AS1 or control siRNA for 36 h and then subjected to flow cytometry cell cycle and apoptosis assays. Relative to the scrambled control-siRNA-transfected cells, the AFAP1-AS1 knockdown had no significant effect on cell cycle distribution or apoptosis.

After establishing siRNA efficacy, we assessed the phenotype changes induced by AFAP1-AS1 knockdown in the NPC cell lines. Wound healing assays demonstrated that the migratory potential of AFAP1-AS1-silenced cells was significantly reduced when compared with scrambled control-siRNA treated NPC cells (Figure 3B). Matrigel invasion assays demonstrated that AFAP1-AS1 siRNA transfected NPC cells had lower invasive capability (Figure 3C). However, AFAP1-AS1 siRNA treatment did not affect cell viability (Figure 4A), cell cycle progression or apoptosis (Figure 4B).

### AFAP1-AS1 knockdown inhibited NPC metastasis in nude mice

**Figure 5:**
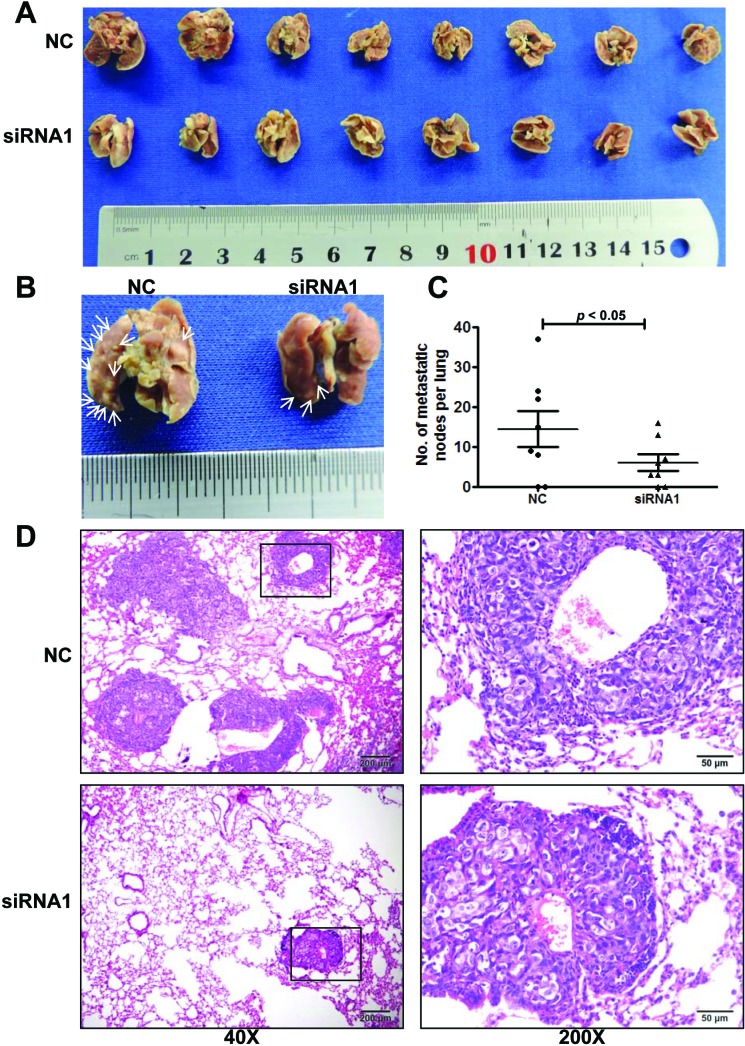
AFAP1-AS1 knockdown inhibited NPC 5-8F cell metastasis *in vivo* **A.** Bright-field images of mouse lungs taken 10 weeks after injection of 1 × 10^6^ AFAP1-AS1 siRNA or negative control treated 5-8F cells into the tail vein. **B.** Representative images of visible nodules on the mouse lung surface. Arrows indicate clusters of tumor cells that have colonized the lung. **C.** Numbers of visible lung metastases in nude mice. The data are presented as the means ± SEM (each data point represents a different mouse; *n* = 8 mice per group). **D.** Representative images of AFAP1-AS1 siRNA expressing 5-8F cells after metastasis to lung tissue. H&amp;E-stained sections of lung tissues after the mice received injections of AFAP1-AS1 siRNA expressing 5-8F cells. Rectangular boxes indicate clusters of micro metastatic cells in the lung. Images were acquired at 40X and 200X. Scale bars = 200 µm and 50 µm.

To confirm the effects of AFAP1-AS1 knockdown *in vivo*, we inoculated 5-8F cells expressing AFAP1-AS1 siRNA or scrambled control-siRNA into the tail veins of nude mice and assessed the number of metastasized tumor nodules in the mouse lung. AFAP1-AS1 siRNA significantly reduced the size and number of metastasized tumor foci (Figure 5A and 5B). Tumor nodules were detected on the surface of the lung in six of eight mice inoculated with 5-8F control-siRNA cells, and an average of 14.4 ± 4.5 nodules were recorded per mouse. In contrast, lung surface tumor nodules were also detected in six of eight mice inoculated with 5-8F AFAP1-AS1-siRNA cells, but a significantly reduced average of 6.0 ± 2.1 nodules were recorded per mouse (Figure 5C). Hematoxylin and eosin (H&amp;E) staining of paraffin-embedded lung tissues also showed a decrease in the number and size of the metastatic foci in mice inoculated with 5-8F AFAP1-AS1-siRNA cells (Figure 5D).

### AFAP1-AS1 knockdown increased AFAP1 protein levels

**Figure 6:**
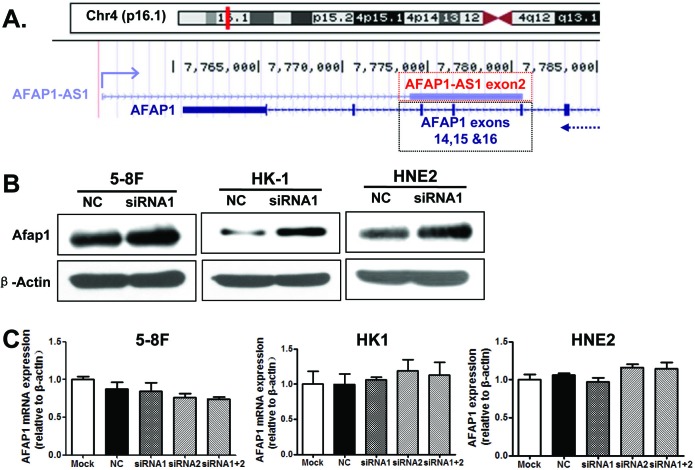
AFAP1-AS1 knockdown upregulated the expression of AFAP1 protein **A.** Alignment of AFAP1-AS1 with the protein-coding AFAP1 gene on chromosome 4p16.1. Three exons (14, 15 and 16) of AFAP1 complemented the second exon of AFAP1-AS1. **B.** AFAP1-AS1 knockdown upregulated AFAP1 protein levels in 5-8F, HK1 and HNE2 cells when compared to the negative control (scrambled control-siRNA). **C.** AFAP1-AS1 knockdown did not affect AFAP1 mRNA levels in 5-8F, HK1 or HNE2 cells when compared to the negative control (scrambled control-siRNA).

*AFAP1-AS1* is localized at the antisense chain of the gene coding AFAP1 protein, and there are overlapping and complementary regions between the second AFAP1-AS1 exon and AFAP1 exons 14, 15 and 16 (Figure 6A). Therefore, we suspected that AFAP1-AS1 might promote cancer cell migration and invasion by interfering with AFAP1 expression. AFAP1 protein levels were elevated after AFAP1-AS1 knockdown in the 5-8F, HNE2, HK1, and A549 cells lines (Figure 6B); however, AFAP1 mRNA expression was not significantly altered (Figure 6C). This suggested that AFAP1-AS1 might regulate *AFAP1* translation or increase the half-life of AFAP1 protein, but that AFAP1-AS1 did not affect *AFAP1* transcription.

### AFAP1-AS1 knockdown induced the loss of cancer cell stress filament integrity

**Figure 7:**
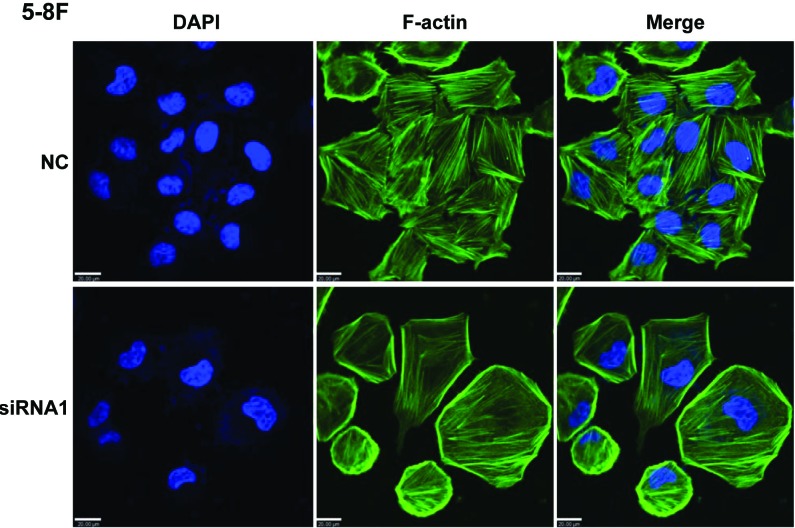
AFAP1-AS1 knockdown induced loss of stress filament integrity in cancer cells Immunofluorescence staining of 5-8F cells after AFAP-AS1 knockdown. Forty-eight hours after the plating of cells onto fibronectin-coated glass coverslips, cells were fixed and stained for F-actin with phalloidin. A clear deficiency in stress fiber formation was observed in AFAP-AS1 knockdown cells. Images were acquired at 400X. Scale bar = 20 µm.

Because AFAP1 is thought to be a modulator of actin filament integrity [20], we investigated the consequences of AFAP-AS1 knockdown on actin filament integrity. No morphological differences were immediately apparent by phase contrast microscopy analysis in the presence or absence of AFAP-AS1 (data not shown); however, although cortical actin staining was still detectable, immunofluorescence analysis demonstrated that, unlike scrambled control-siRNA treated 5-8F cells, phalloidin was unable to decorate stress filaments in cells after AFAP-AS1 knockdown. This indicated that stress filament integrity was lost after AFAP-AS1 knockdown (Figure 7).

### Identification of differentially expressed proteins in AFAP1-AS1 knockdown cells

To identify the proteins or signaling pathways regulated by AFAP1-AS1 expression, we performed proteomics analysis of 5-8F cells transfected with AFAP1-AS1 siRNA or scrambled control-siRNAs using liquid chromatography-tandem mass spectrometry (LC-MS/MS). Among the 209 proteins that were differentially expressed after AFAP-AS1 knockdown, 133 were upregulated and 76 were downregulated (Supplemental Table S3).

**Figure 8:**
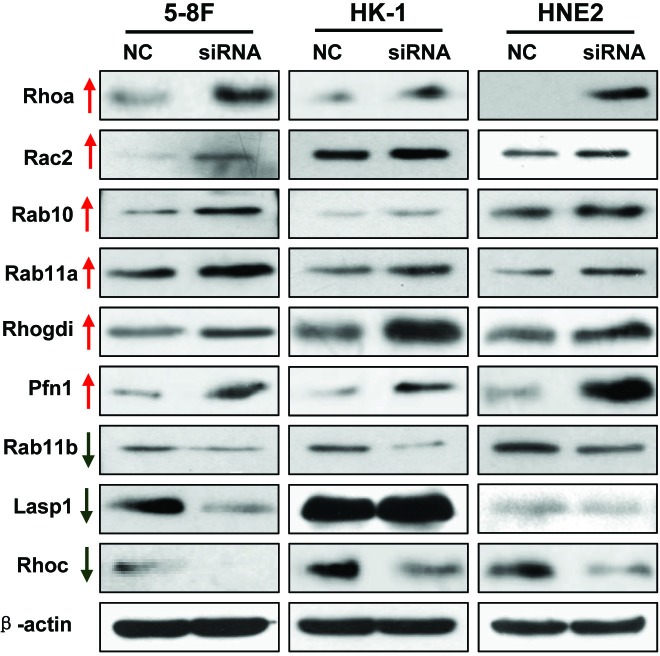
Validation of differentially expressed proteins identified by proteomics Differentially expressed proteins were confirmed by using Western blotting in 5-8F, HK1 and HNE2 cancer cell lines transfected with the scrambled control-siRNA or AFAP1-AS1 siRNA. The results were consistent with the proteomic analysis.

DAVID Bioinformatics software analysis indicated that many cytoskeletally-regulated proteins were significantly altered after AFAP-AS1 knockdown, including proteins in the small GTPase signaling Rho/Rac pathway. We confirmed these results *via* Western blotting analysis of tumor cells expressing AFAP1-AS1 siRNA, and found that RhoA, Rac2, Rab10, Rab11a, Rhogdi and Pfn1 were significantly upregulated, but RhoC, Rab11b and Lasp1 were significantly downregulated in NPC cell lines (Figure 8).

## DISCUSSION

Many studies have reported a close association between lncRNA expression and tumor development and progression [21-23]. In the current study, we first profiled the differential expression of genes in 12 NPC and 4 NPE tissue samples, and combined our data with a previously published GEO dataset (GSE12452) [19] to identify differentially expressed lncRNAs in NPC. We found 24 differentially expressed lncRNAs in NPC tissues when compared with the non-cancerous NPE tissue samples. Among these 24 lncRNAs, we chose and focused on AFAP1-AS1, which only been previously reported in esophageal cancer [24]. We performed qRT-PCR and *in situ* hybridization to verify our results using existing data in the GEO database. We found AFAP1-AS1 was upregulated in NPC and its upregulation was associated with tumor progression and poor survival. We then performed *in vitro* and nude mouse experiments to confirm our *ex vivo* data. Our *in vitro* experiments demonstrated that AFAP1-AS1 knockdown inhibited the invasive and migratory capacity of NPC cell lines and nude mouse experiments confirmed that AFAP1-AS1 knockdown suppressed NPC cell metastasis to mouse lungs. Furthermore, proteomic analysis indicated that AFAP1-AS1 could regulate the expression of small GTPases and NPC cell stress filament integrity.

Previous studies have demonstrated that lncRNA activity was partly dependent on genomic location. Antisense lncRNAs like AFAP1-AS1 are oriented in an antisense direction with respect to a protein-coding gene in the opposite strand and usually act as a regulator of this gene [25-28]. AFAP1-AS1 is localized in the antisense DNA strand of the *AFAP1* gene. We demonstrated that AFAP1-AS1 expression increased AFAP1 protein levels, but did not affect *AFAP1* mRNA levels. But it remains unknown whether the effects of AFAP1-AS1 on regulation of tumor cell metastasis potential are mediated by the changed AFAP1 protein levels. AFAP1 is an adapter molecule to link to other proteins, such as SRC and PKC, to modulate changes in actin filament integrity and induces lamellipodia formation [29, 30]. Interestingly, our current *in vitro* data demonstrated that AFAP1-AS1 knockdown led to a loss of stress fiber formation. This suggests that AFAP1-AS1 might promote tumor metastasis through regulation of tumor cell adhesion and mobility. We found more than 200 differentially expressed proteins to be targeted by AFAP1-AS1, including Rho/Rac GTPase family members and actin cytokeratin signaling pathway proteins. In mammalian cells, small GTPases regulate actin cytoskeleton formation and generation of an actin-based dynamic motile structure and cytoskeletal control of cell shape and migration. Cancer cells acquire migratory and invasive properties through disruption of cell-cell junctions, changes in focal adhesion complexes and extensive reorganization of the actin cytoskeleton. AFAP1-AS1 might modulate changes in actin filament integrity through AFAP1 gene. Therefore, future studies should investigate whether this molecule can be targeted as part of a strategy to control tumor progression.

Previous studies have demonstrated that lncRNA regulates gene transcription and post-transcriptional modification or translation and altered expression of lncRNA associated with human carcinogenesis [31-33]. A recent study reported that only one-fifth of the transcription activity in the human genome is translated into proteins [34-36], indicating that there are at least four times as many non-coding as there are coding sequences in the human genome. But there were few reports on lnRNA alterations in NPC. In the current study, we identified 24 differentially expressed lncRNAs in NPC including GAS5 [37, 38], NEAT1 [39, 40] and H19 [41-44], which contribute to the development and progression of cancers and might serve as novel, non-invasive biomarkers for cancer diagnosis, progression and prognosis.

In conclusion, we demonstrated and confirmed the upregulation of AFAP1-AS1 in NPC. AFAP1-AS1 expression was associated with poor prognosis, suggesting that AFAP1-AS1 may be a useful biomarker for the prediction of NPC progression and prognosis. We also found that that AFAP1-AS1 knockdown significantly suppressed the invasive and metastatic abilities of NPC cells, indicating that further investigation of AFAP1-AS1 may lead to the development of novel NPC therapies.

## MATERIALS AND METHODS

### Tissue samples

Three sets of NPC samples were collected for this study: Set 1, tissue biopsies for gene profiling, contained 12 NPC and 4 NPE tissue samples for Affymetix HG_U133 Plus 2 microarray analysis; Set 2, contained 23 primary NPC and 7 non-tumor NPE biopsies to verify AFAP1-AS1 expression with qRT-PCR; and Set 3, included 112 paraffin-embedded NPC and 10 NPE tissue samples for *in situ* hybridization confirmation of AFAP1-AS1 expression. All tissue samples were collected from newly diagnosed NPC patients at the Xiangya Hospital and the Affiliated Cancer Hospital of Central South University (Changsha, China). All specimens were confirmed by histopathological examination. All of these patients have received routine radiotherapy. This study was approved by the hospital Research Ethics Board of Xiangya Hospital and the Affiliated Cancer Hospital of Central South University, and signed informed consent was obtained from each participant before they were enrolled in the study. Clinicopathological data were collected from patient medical records and are reported in Supplemental Tables S2 and S4.

### RNA isolation, amplification and microarray hybridization

RNA isolation and amplification were performed as described previously [45]. Briefly, we performed laser capture microdissection (LCM) to enrich tumor cells from NPC tissue sections and isolate the epithelial compartment from non-tumor nasopharyngeal tissue specimens [46]. RNA was isolated from LCM-enriched tissues using an RNeasy Mini Kit (Qiagen, Hilden, Germany) and reverse transcribed into cDNA. Half of the cDNA was used according to manufacturer's protocols as a template for bacteriophage T7 RNA polymerase to synthesize biotinylated antisense RNA for hybridization to the Affymetrix Human Genome U133 Plus 2.0 oligonucleotide microarrays (Affymetrix, Santa Clara, CA, USA). All microarray expression data were deposited in the Gene Expression Omnibus (GEO) database under accession number GSE64634.

### Data analysis

After obtaining our own gene expression data, we also downloaded another NPC gene expression data from Affymetrix Human Genome U133 Plus 2.0 platform based studies in the GEO database (accession number GSE12452) [19]. Our data analysis procedures are shown in Figure 1A. Significant Analysis of Microarray (SAM) software [47] were used to analyze normal nasopharyngeal epithelium and NPC tissue samples for differences in lncRNA expression in our data (GSE64634) and a published NPC dataset (GSE12452 [19]). The cut-off value for differentially expressed lncRNA was set at ≥ 1.5-fold difference and the false discovery ratio (FDR) was < 0.05.

### Cell lines and siRNA

NPC cancer 5-8F, HNE2 and HK-1 cell lines were maintained in our laboratory. Cells were cultured in RPMI 1640 medium supplemented with 10% fetal bovine serum (FBS, Invitrogen, Shanghai, China), penicillin (100 U/ml, #P3032, Sigma Chemicals, St Louis, MO, USA) and streptomycin (100 g/ml, #WB11000, Sigma Chemicals) in a humidified incubator with 5% CO_2_ at 37°C. For gene knockdown, cells were seeded overnight and transfected with either 50 nM siRNA or non-target scramble control siRNA (Invitrogen) using Lipofectamine RNAiMAX Reagent (Invitrogen, Breda, The Netherlands) in OptiMEM medium (Invitrogen). The sequences of the AFAP1-AS1 targeting siRNAs were 5′-GGGCTTCAATTTACAAGCATT-3′ and 5′-CCTATCTGGTCAACACGTATT-3′. Sequences of non-target scramble controls were provided by Invitrogen.

### Wound healing assay

Cells were seeded and grown to 90% confluence in 6-well culture plates. A p200 pipet tip was used to create a scratch in the cell monolayer. Images were captured 0, 24 and 48 hours after wounding. Wound width was measured with an ocular ruler to ensure that all wounds were the same width at the beginning of each experiment.

### Matrigel invasion assay

Tumor cell invasion capacity was assessed using Transwell Cell Culture Inserts (8 µm pore size, BD Biosciences, New Jersey, USA) in 24 well plates. A total of 1 × 10^5^ cells in 100 µl of serum-free medium were added to the top chamber. The bottom well contained growth medium with 20% FBS. Cells were incubated for 36 h at 37°C and then the cells that had invaded through the filter pores were fixed with methanol, stained with hematoxylin and observed under a microscope. The number of invasive tumor cells were counted from five randomly selected 20 × fields for each experiment and averaged.

### Cell viability MTT assay

Changes in cell viability were determined by adding 20 µl of 1 mg/ml tetrazolium salt (3-(4, 5-dimethylthiazol-2-yl)-2, 5-diphenyltetrazolium bromide, MTT) reagent (Sigma Chemicals, St Louis, MO, USA) to each well at 0, 1, 2, 3, 4 and 5-day time points. Experiments were performed in triplicate and repeated once.

### Flow cytometry cell cycle and DNA content analysis

Cell cycle distribution and apoptosis were measured *via* flow cytometry. DNA content was detected using PI staining 48 h after siRNA transfection according to the methods described in a previous study [48]. This assay was repeated once and conducted in triplicate.

### qRT-PCR

qRT-PCR was performed using a SYBR_Premix ExTaq II kit (Takara, Dalian, China) in the CFX96 Real-Time PCR Detection System (Bio-Rad, Hercules, CA, USA) to determine the relative expression levels of target genes. The primers used were AFAP1-AS1, 5′-AATGGTGGTAGGAGGGAGGA-3′ and 5′-CACACAGGGGAATGAAGAGG-3′; AFAP1, 5′-AGAGTGTCCTCCTCCACCAA-3′ and 5′-CTTGGCCTCTGATTTGGAAC-3′; ACTB (β-actin), 5′-TCACCAACTGGGACGACATG-3′ and 5′-GTCACCGGAGTCCATCACGAT-3′. ACTB was used as the reference and normalization control. The average of three independent analyses for each gene was calculated.

### Proteomic analysis

To screen molecules down-stream of AFAP1-AS1, we performed proteomic analysis after AFAP1-AS1 knockdown using an Ultimate 3000 RSLCnano system coupled to an LTQ Orbitrap Velos Pro mass spectrometer (Thermo Scientiﬁc, Bremen, Germany). Peptide was diluted with 0.1% trifluoroacetic acid (TFA) and 5 μl sample was injected for each analysis. Next, the analyte was transferred to the analytical column. The mass spectrometer was operated in a data-dependent mode. Full scan MS spectra were acquired at a mass resolution of 60,000 (mass range 350 - 2000 m/z) in the Orbitrap analyzer. For label-free analyses, tandem mass spectra of the ten most abundant peaks were acquired in the linear ion trap by peptide fragmentation using collision-induced dissociation (CID). Normalized collision energy (NCE) was set to 35% and an isolation width of 2 m/z was chosen [49, 50].

Proteins were identified using Proteome Discoverer software (Thermo Scientific, Waltham, MA, USA). Thermo raw files were imported and used to conduct a search of the UniProt KB/Swiss-Prot database (release 2014_02). For database searches, mass tolerances were set to 10 ppm and 0.8 Da for precursor and fragment ions, respectively. Peptide identification with false discovery rates < 1% (q-value < 0.01) were discarded. Proteins that met the following criteria were considered differentially expressed proteins: i) proteins were identified based on ≥ 2 peptides with ≥ 95% confidence and ii) protein levels demonstrated an averaged ratio-fold change of ≥ 1.50 or ≤ 0.67 (Student's *t* test, *p* < 0.05) in the LC-MS/MS analyses. DAVID Bioinformatics software (http://david.abcc.ncifcrf.gov/summary.jsp) was used to determine the signaling pathways altered after AFAP1-AS1 knockdown.

### Immunofluorescence

Cells were fixed in 4% paraformaldehyde for 20 min, permeabilized with 0.5% Triton X-100 for 3 min and blocked in phosphate-buffered saline (PBS) containing 7% fetal bovine serum for 30 min. Cells were then incubated with Alexa Fluor 488 phalloidin (Molecular Probes, Eugene, OR, USA) for 1 h. After incubation, cells were washed three times with PBS, then incubated with 49, 6-diamidino-2-phenylindole (DAPI) for 10 min at room temperature. Immunofluorescence images were collected using a confocal fluorescence microscope (UltraView Vox; PerkinElmer, Waltham, MA, USA).

### Western blotting

Lysis, electrophoresis and target protein visualization were performed as described previously [8]. Briefly, 50 μg of cell lysates were separated by 10% sodium dodecyl sulfate-polyacrylamide gel electrophoresis (SDS-PAGE), and then transferred onto a PVDF membrane. Membranes were incubated overnight at 4°C with primary anti-RhoA, RhoC, Rac2, Rab10, Rab11b, Rab11a, Rhogdi, Pfn1 or Lasp1 antibody (Proteintech, Wuhan, China). The next day, blots were washed with PBS and then incubated with a horseradish peroxidase-conjugated secondary antibody for 1 h at room temperature. The signal was visualized using an ECL detection reagent and quantified by densitometry using Image J software (http://rsb.info.nih.gov/ij). β-Actin was used as a loading control, and was detected using mouse anti-β-Actin antibody (Proteintech, Wuhan, China).

### *In situ* hybridization

*In situ* hybridization was performed to detect AFAP1-AS1 expression in tissue specimens using three 30-base nucleotide probes from different *AFAP1-AS1* regions. The AFAP1-AS1 probes were5′- ATTCCTTTATTTTATGGGATGTTCTGTAGGGAGTT-3′,5′-TAGAAATGAGAAAAGAATCACCAAGAGAGTAAGCA-3′, and 5′- CCCTACAGCTAGTTTCCTCTTCATTTATTCATTT-3′. Three GAPDH probes used as positive controls were 5′-CCACTTTACCAGAGTTAAAAGCAGCCCTGG-3′, 5′-CAGTAGAGGCAGGGATGATGTTCTGGAGAG-3′, and 5′-GTCAGAGGAGACCACCTGGTGCTCAGT GTA-3′. The probes were synthetized and labeled with DIG-dUTP at the 3′ end using a kit from Invitrogen (Shanghai, China). *In situ* hybridization was performed as previously described [45]. A semi-quantitative scoring criterion for *in situ* hybridization was used in which both the staining intensity and the number of positive cells were recorded. All sections were independently scored by two pathologists who were blinded to the clinicopathological features and the clinical data.

### Animal experiments

To confirm the role of AFAP1-AS1 in the regulation of cancer cell invasion and metastasis, we performed nude mouse tail-vein injections of tumor cells. Sixteen mice were divided into two groups. The AFAP1-AS1 or scrambled control-siRNA transfected 5-8F cells (1 × 10^6^ cells) were washed once with PBS and then injected into the tail vein of four-week-old nude mice. All mice were sacrificed 10 weeks after inoculation. Lymph nodes, lung, pancreas and liver tissues were removed from each mouse, weighed, photographed, embedded in 10% paraffin and subjected to sectioning and H&amp;E staining for histological examination and evaluation of metastasis. The numbers of visible lung-surface metastases in each mouse were recorded. Animal experiments followed protocols approved by the Institutional Animal Care and Use Committee of Central South University (Changsha, China).

### Statistical analysis

Statistical analysis was performed using SPSS software, version 19.0 (SPSS, Chicago, IL, USA). Student's *t*-tests were used to evaluate significant differences between any two groups of data and one way ANOVA were used to evaluate significant differences for multiple comparisons. Overall survival (OS) or relapse-free survival (RFS) were calculated using the Kaplan-Meier method, and the results of the analysis were considered significant in a log-rank test if *p* < 0.05. All data are represented as means ± standard deviation. Differences were considered significant if *p* < 0.05.

## SUPPLEMENTARY TABLES










