# Supplementary material for: Upregulated long non-coding RNA AFAP1-AS1 expression is associated with progression and poor prognosis of nasopharyngeal carcinoma
Source: Oncotarget. 2015 May 9;6(24):20404–18. doi: 10.18632/oncotarget.4057 (PMC4653014; doi:10.18632/oncotarget.4057)
Supplement: Supplementary file 1 [file oncotarget-06-20404-s001.pdf]

Upregulated long non-coding RNA AFAP1-AS1 expression is associated with progression and poor prognosis of nasopharyngeal carcinoma

Supplementary Material

Supplemental Table S1. Significantly dysregulated lncRNAs in the two NPC GEO datasets (GSE13452 and GSE64634) with SAM analysis

Supplemental Table S2. Clinicopathological data for 112 paraffin-embedded NPC biopsies and the AFAP1-AS1 expression levels of these samples as measured by *in situ* hybridization

Supplemental Table S3. AFAP1-AS1 regulated proteins in 5-8F cells identified by LC-MS analysis

Supplemental Table S4. Clinicopathological data for the 35 primary NPC biopsies and 11 NPE tissues used for gene profiling and qRT-PCR
